# Assessment of complex projectiles in the early Late Pleistocene at Aduma, Ethiopia

**DOI:** 10.1371/journal.pone.0216716

**Published:** 2019-05-09

**Authors:** Yonatan Sahle, Alison S. Brooks

**Affiliations:** 1 DFG Center for Advanced Studies: “Words, Bones, Genes, Tools”, University of Tübingen, Germany; 2 Center for the Advanced Study of Human Paleobiology, Department of Anthropology, The George Washington University, Washington, DC, United States of America; 3 Human Origins Program, National Museum of Natural History, Smithsonian Institution, Washington, DC, United States of America; Max Planck Institute for the Science of Human History, GERMANY

## Abstract

Complex projectiles—propulsion via mechanical aid—are considered an important technological innovation, with possible relevance for the successful Out-of-Africa dispersal of our species. Conclusive evidence for the beginning of this technology, however, is lacking from the *early* Late Pleistocene (ca. 130 to 70 thousand years ago; ka). Given the extremely limited applicability of relatively robust methods for validating stone-tipped projectile use, such as through fracture propagation velocity, converging lines of circumstantial evidence remain the best way to examine early complex projectiles. We assess here suggestions for an early Late Pleistocene origin of complex projectiles in Africa. Results from both previous and present independent approaches suggest a trajectory in which complex projectiles were likely adopted during the early Late Pleistocene in eastern Africa. At Aduma (Middle Awash, Ethiopia), morphometric, hafting, and impact damage patterns in several lithic point assemblages suggest a shift from simple spear technologies (thrusting and/or hand-cast) to complex projectiles. Broadly dated to 80–100 ka, lithic points from later phases of the Aduma succession represent a particularly strong candidate for projectile armatures most comparable to ethnographically known spearthrower darts, lending support for previous suggestions and warranting further investigations.

## Introduction

Complex projectiles, such as the bow-and-arrow, and spearthrower-and-dart, create greater acceleration and hence confer extensive impact range and better accuracy than hand-cast spears, or simple projectiles. Such enhanced ability to wound/kill at greater distance also reduces the danger involved in hunting and violent interpersonal encounters. Because complex projectiles allow exploitation of broader niches, and because they are not known from any non-*sapiens* context, they are considered an “enabling technology” [1] that may have played a pivotal role in the expansion of early *H*. *sapiens* [2]. Complex projectiles require combining independent tool components into a single hunting device. Their emergence in the archaeological record is, therefore, considered to have significant cognitive implications as well [2,3]. Over the past decade, African archaeological assemblages with potential early complex projectiles have received greater research attention (e.g.,[2,4–7]). However, with strong evidence for such armatures as yet unknown from the early Late Pleistocene (i.e. prior to ~70 ka), a clear understanding of their development remains a challenge.

Based on overall dimensions, shapes, weights, macro-fracture patterns, hafting traces, and analogy with ethnographic and experimental hunting armatures, several assemblages of pointed artifacts from sub-Saharan Late Pleistocene archaeological occurrences have been suggested to represent possible early complex projectiles [2,6,7]. Unfortunately, each of these suggestions made on the basis of the aforementioned attributes suffers from several limits of inference [8]. Identifying one or more of the outlined lines of evidence surely can hint at the potential function of certain stone points as tips of hunting weapons. However, confidently establishing the actual armature type or mode of delivery requires direct evidence such as actual spear hafts, bows or spear throwers, arrow or dart shafts, or images of same, all of which are thus far lacking from the sub-Saharan early Late Pleistocene archaeological record. As a result, unlike evidence from the recent past, for which careful relational analogies may provide more reliable information about the actual armature type and/or delivery mechanism [9], identifying projectiles of great antiquity remains extremely difficult.

*Early* Late Pleistocene points from localities at Aduma have been suggested—on the basis of treatment of the proximal end, consistency of point angle, and progressive reduction in size and weight across the succession—to be evidence for the adoption of early complex projectiles by ~80 ka [2]. However, analytical approaches in Brooks *et al*. [2: 244] differ from what have been employed in similar investigations in that the former involved “a total of 25 variables describing attributes of the blank, the divergence and shape of the sides, point asymmetry, marginal retouch, and treatment of the base.” This methodological distinction rendered subsequent comparative assessment of the Aduma inferences difficult [5].

Because the Aduma assemblages sample a critical time period for studying the evolution of complex projectiles, and because some of these appear in stratigraphic association with the remains of anatomically modern humans [10], we assess here inferences in Brooks *et al* [2] using the more commonly employed impact fracture [11–14], and tip cross-sectional data [4,7,15]. We also employ in the present study independently and directly collected data on previously reported variables, namely point angle, weight, and proximal treatment. Results from these mutually exclusive approaches reveal a pattern in the Aduma archaeological record largely in agreement with previous suggestions for the origin of complex projectiles during the early Late Pleistocene [1,2,4].

### Overview of the current evidence

Claims for early complex projectiles derive from the following sub-Saharan contexts. Cautious interpretation of microwear and residue data on segments and backed pieces from Sibudu Cave (South Africa) posits that these *~*64-ka-old pieces may have served as transversely hafted arrowheads and/or barbs [6,16]. Serrated bifacial points from older (~77 ka) layers at Sibudu have recently been presented as exhibiting evidence of use as projectiles [17]. While the wear and residue evidence on these serrated points seems to show hafting and contact with animal soft tissue, the type of projectile (i.e., simple *vs*. complex) remains difficult to establish. The small sample size of 25 serrated pointed pieces and the lack of clear impact fractures make it difficult to assess the “arguments in favour of bow-and-arrow technology or, alternatively, perhaps flexible spear-throwers (cords)” [17]. Dimensional and morphological attributes for the Sibudu serrated points are difficult to compare with those from other archaeological and ethnographic examples of complex projectiles. A mean length of 41mm, width of 23mm, and thickness of 8mm are reported by Rots et al. [17] for the 25 points and point fragments. These values increase to 44.75mm, 26.83mm, and 8.38mm, respectively, when restricting the counts to complete points only (*n* = 8). If these Sibudu bifacial serrated and hafted points were at all used as projectile tips, they appear to represent spear tips [*cf*. 5], just outside the range of ethnographic dart tips, both in terms of size and tip morphology (calculating tip cross-sectional values from their limited data). Hafted points could be used as knives or thrusting/throwing spears. In this regard, more samples and data are needed to demonstrate that the Sibudu pointed piece were used as high-velocity, complex projectile, rather than hand-cast spears.

Comparably old complex projectiles may have existed following the appearance of “microlithic” technologies ~71 ka at Pinnacle Point [18]. Such suggestions, however, based on the analogy that similar microliths from more recent contexts were probably used to tip arrows and darts, have yet to be assessed using direct analysis of the tools. In a recent moderately exhaustive review of early projectiles, O’Driscoll and Thompson [19] use assemblages from Pinnacle Point 13B (PP13B) to illustrate the potentials of projectile impact marks on archaeofaunal remains to augment inferences about prehistoric hunting armatures. They suggest projectiles, most likely in the form of spear tips, were in use 91–98 ka at PP13B based on a few drag marks on vertebral and rib fragments. However, conclusive results even for the use of simple projectile technology at PP13B must rule out a) potential use of hafted spears as thrusting, instead of projectile, weapons; and b) the possibility of drag marks and embedded quartz tool fragments being the result of carcass processing using points hafted as knives [20], or c) use of bones as percussors, such as is claimed at Sibudu [17]. These possibilities are necessary to consider given aspects of the PP13B lithic data, particularly edge damage, which supports use of the points for cutting activities, rather than as spear tips [21].

Widely studied lithic and faunal assemblages from the Klasies River archaeological record provide another instance of potential early Late Pleistocene projectile use [22]. Evidence of a quartz fragment embedded in the vertebra of an extinct species of buffalo has been interpreted as resulting from hunting with stone-tipped projectiles. However, this interpretation should address the limits of inference identified above for claims from PP13B. In addition, even the smallest tip cross-sectional values for Klasies River lithic assemblages fall outside the range of *complex* projectiles, but rather within those of experimental thrusting spear tips [4].

Late Pleistocene point assemblages from Porc-Epic Cave in Ethiopia have been presented as candidates for early complex projectiles. Based on morphological and metrical attributes, Sisk and Shea [7] suggest that the Porc-Epic unifacial and bifacial points may have effectively functioned as tips of early complex projectiles. Consistent with this suggestion, the Porc-Epic Cave fauna indicates systematic hunting and exploitation [23]. However, these indications require a close investigation of point damage patterns and other relevant attributes; age estimates, the most recent of which center around 50 ka [24], and stratigraphic placements for the Porc-Epic assemblages similarly require reassessment.

**Fig 1:**
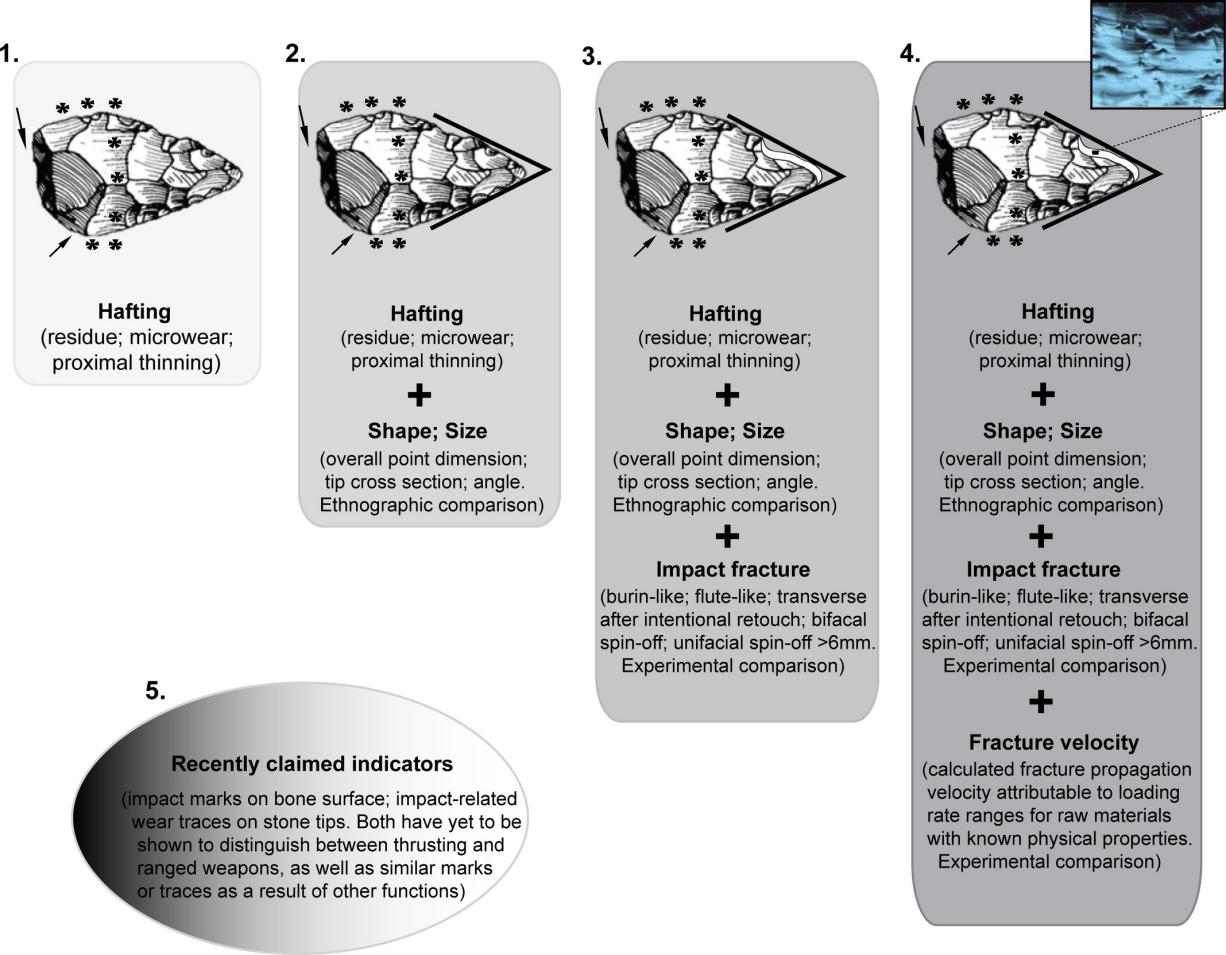
A schematic representation of evidence and methodology widely used for determining prehistoric projectile weaponry. Strength of evidence increases from ***1*** (i.e. hafting) to ***4***. Larger ethnographic datasets and more experiments are still needed to test these indices further. Impact fractures considered diagnostic of projectiles do not distinguish between *simple* and complex projectiles; more work should explore this possibility. Methods depicted in ***5*** require adequate demonstration of applicability. In all approaches, the claim for projectile use should effectively eliminate potential use as thrusting spears, or even as hafted knives, barbed clubs, etc.

To summarize, the existing evidence for the development of complex projectiles prior to 40 ka is far from clear. Direct evidence for such weapon systems is to date unknown from this period, while hardly any lithic assemblage from the various sub-Saharan contexts has received multi-stranded investigation. Each approach to the study of prehistoric weaponry has its own interpretive shortfalls. For instance, Sisk and Shea [7] clarify that the cross-sectional area and -perimeter (TCSA and TCSP) of a pointed artifact’s tip only assess its suitability to function as an effective projectile. Similarly, remarks have been made about direct evidence for hafting of pointed pieces not being adequate to making a case about the function and delivery mode of “the hafted” stone artifact [8]. Impact fractures of a certain nature provide a quick means to assess prehistoric projectile technologies. However, such diagnostic impact fractures (DIFs) alone cannot differentiate between simple and complex projectiles, and are therefore not categorical [14]. The same can be said about microwear methods. Impact marks on bony remains from archaeological contexts promise a new avenue for investigating prehistoric weaponry and foraging adaptation, if proven to distinguish between projectile and non-projectile inflicted lesions [19]. Ultimately, only a combination of results emanating from several independent approaches and/or direct conclusive evidence promise convincing inferences about the beginning of early projectiles (Fig 1).

Point size and morphology arguably represent important variables that prehistoric hunters optimized to meet hafting requirements as well as achieve the desired aerodynamic and penetrative abilities of hafted points. Recently, Newman and Moore [9] have shown that the morphometric approach based on a point’s tip cross-sectional area (TCSA) and -perimeter (TCSP) is not applicable to ethnographic Australian spearthrower darts. Similarly, experimental study by Clarkson [25] suggests that there is very poor correlation between TCSA/TCSP and the penetration depth of projectile points. A more recent experiment [26], however, has demonstrated that TCSP indeed significantly correlates with—and is hence a strong predictor for—penetration depth. Unlike Grady [26], the robustness of TCSP alone has not been thoroughly evaluated in Clarkson [25]. Sisk and Shea [7: 6] remark that “it is possible that the ethnographic controls themselves do not fully capture the variability in complex projectile point morphology”. Given the uniqueness of the Australian fauna—both prey and predator species—compared to Africa and the Americas, it will be interesting to see if prehistoric and ethnographic hunter-gatherers optimized point tip morphology and size differently. An additional caution is due to the fact that the organic elements of hafting and propulsion weapon systems are rarely preserved in Pleistocene or early Holocene sites. Consequently the relationship between ethnographic weapons and prehistoric ones may be even more ambiguous. This is particularly true when we only have the stone weapon armatures, as the archaeological population may have used several different weapon systems, much as ethnographically known people did, but the pointed armatures may look similar. Residues may provide clues to how the point was originally hafted, but not necessarily to the weapon system itself.

Macroscopic fracture types most commonly considered diagnostic of impact from use of pointed pieces as weapon tips include: i) burin-like fractures; ii) flute-like fractures; iii) transverse fractures with terminations other than snaps that occurred after the artifact was retouched; iv) bifacial spin-off fractures; v) unifacial spin-off fractures with a fracture length of >6mm [11]. Impact fracture initiations are commonly bending, rather than cone—where the cone of force leaves a distinctive bulb [27]. Similarly, fractures that retain negative bulbs of percussion, and those with feather terminations are often indicative of a manufacturing process, rather than impact damage [*cf*. 28]. Such fractures were treated in this study cautiously (for instance, with attention to the sequence of fractures; [13]).

Impact fractures are not exclusively limited to the distal tips and distolateral portions of pointed pieces; they can sometimes occur along the medial/proximal portion of a pointed piece where an impact creates a transverse fracture [29]. Additional lines of evidence suggestive of hafted weapon use include treatment of the proximal end of pointed pieces to facilitate hafting, and ventral flaking of the distal tip [2,30].

Impact-induced fractures on cryptocrystalline and amorphous materials often form primary Wallner lines: velocity-dependent ripple marks created when a crack force encounters intrinsic imperfections in the rock [31]. Using the physical properties of the raw material and the geometry of Wallner lines, it is possible to calculate the instantaneous velocity of an impact fracture and the probable delivery mechanism responsible for producing the specific precursory loading. Although this method should theoretically work in all fine-grained raw materials, the features are most clearly documented in obsidian [32,33]. Still, even for such high-quality raw materials with clear microfracture features as obsidian, inferences from this approach require knowledge about the physical properties of the specific source rock (such as the Young’s Modulus and specific gravity) on which the artifacts of interest are made.

### Background to the Aduma archaeology

The archaeological occurrences in the Aduma area of the Middle Awash research project were closely investigated during much of the 1990s. Excavations and controlled surface-collections, conducted at several localities and under the directorship of John E. Yellen and one of the present co-authors (A.S.B), have yielded more than 16,000 artifacts, including some in spatial association with faunal as well as hominid remains [34]. Archaeological finds at Aduma derive from sediments today exposed by erosion, and postdate occurrences containing large core tools in stratigraphically lower gravel lags.

Four of the excavated localities with *in situ* archaeology (namely, Aduma A1, A4, A5, and A8) were subsequently examined in detail by Yellen, Brooks and colleagues [34]. Aduma A1 represents the oldest of these localities, occurring immediately above an erosional surface and below the undated Bodole tuff at the base of a unit designated as “Ardu B”. An ^40^Ar-^39^Ar date of 180 ka on reworked pumices provides a maximum age constraint for all of the archaeological occurrences in the Ardu B sediments. The Ardu B sediments containing the Aduma A4, A5, and A8 localities have yielded age estimates from various techniques (primarily U-series on mammal teeth and fossil bone, and OSL), centering on a range of ~80–100 ka. The most reliable of these are OSL determinations between 91 and 93 ka (*Table 2* and *discussion* in Yellen *et al*. [34]).

**Fig 2:**
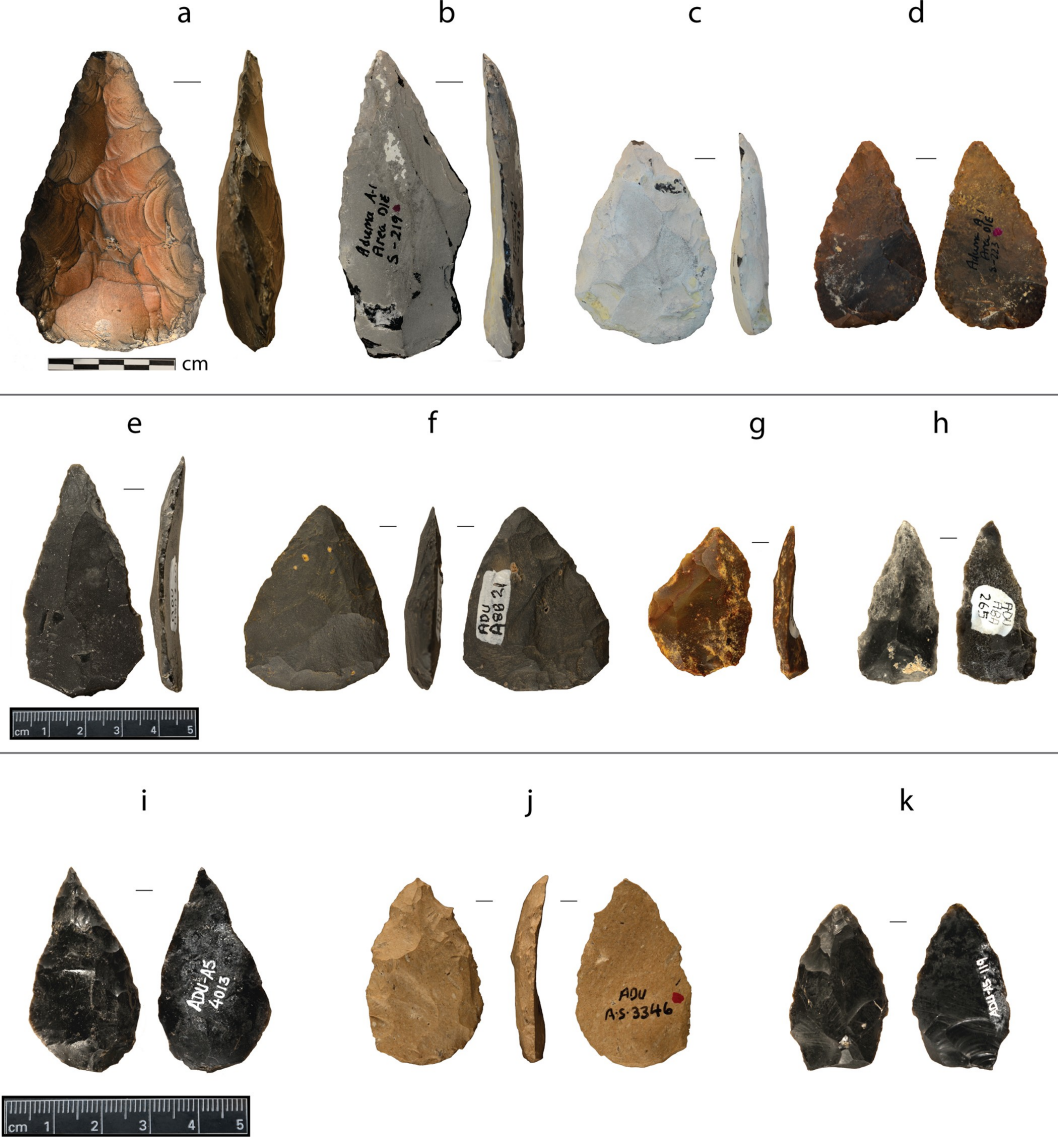
A sample of pointed artifacts from Aduma: A1 diminutive handaxe and points (top row); A4 and A8 points considered intermediate in age (middle row); and A5 points (bottom row). (*a*) small, triangular handaxe on obsidian; (*b-k*) points. Note that items *b*, *e*, *h*, and *i* have acute-tip; *c* and *f* broad-base; *d*, *e*, *f*, *h*, *i*, and *k* proximal retouch; and *a*, *b*, *c*, and *h* heavy patination.

The basal Aduma A1 assemblage differs in several ways from the remaining three younger localities in the overlying Ardu B sediments. Although much of the Aduma A1 material was recovered as surface collection, a small area was excavated into the base of the silt overlying the unconformity in order to control for potential deflation and mixing in the surface assemblage [2]. The excavated Aduma A1 assemblage is dominated by variants of Levallois technology. The cores and pointed pieces do contain types that are common in the upper Ardu B localities. However, the A1 assemblage differs from those of the younger Aduma localities in the presence of small bifaces, and large points on Levallois flakes (Fig 2). Still, Brooks *et al*. [2: 242] note that the presence in the Aduma A1 assemblage of certain artifact “types identical to those found higher in the sequence could signal stratigraphic admixture”.

The upper Ardu B localities at Aduma (i.e., A4, A5, and A8) share a number of typological similarities: “classic”, “acute tip”, and “short broad” points dominate all three assemblages (*sensu* [2]; Fig 2). Aduma A5, stratigraphically the youngest, stands out from the others by the unique presence of what the original excavators termed as “small blunt points” as opposed to “short, broad” ones which are found at all the Ardu B sites [34] (Fig 2). The stratigraphic relationship between A4 and the A8 localities has been more difficult to determine with certainty as the two areas are separated by ~4 km of variably dissected and aggraded sediments.

## Materials and methods: Identifying archaeological complex projectiles

For the present study, the TCSA/TCSP method is employed along with data on artifact dimension, weight, point angle, proximal thinning, and DIFs. A total of 122 pointed artifacts were analyzed for this study from the Aduma A1, A4, A5, and A8 localities. All studied specimens are housed in the National Museum of Ethiopia, Addis Ababa; permissions for this study were obtained from the Authority for Research and Conservation of Cultural Heritage (Addis Ababa) and the Middle Awash project leadership.

Aduma A8 points included in this study comprise assemblages from the main A8 and A8A excavations—as well as one piece from A8B. Aduma A8A and A8B are located 50-100m from the main A8 excavation in different directions. To the east of A8, Aduma A8A represents a small excavation, and surface collection, considered to be a lateral extension of the artifact level at the main A8 locality [34]. To the west of the main excavation, Aduma A8B was a deflated hippo butchery occurrence that was sieved.

This study has excluded pointed pieces that were a) too fragmentary on which to collect any of the suitable measurements, and b) categorized under a different tool class, rather than hunting weapon tips, during reanalysis. The latter includes a small, bifacially worked triangular handaxe (length = 11.75cm; max. width = 7.42cm; max. thickness = 2.54cm) made on obsidian, and several pieces in the “point-perforator/borer” category of Yellen *et al*. [34]. The exclusion of these artifacts is meant to enable a more conservative assessment with pointed pieces more likely to have been used as projectile tips (probable hunting weapon tips are limited to pieces that lack clear becs created by adjacent notches, and retain damage patterns consistent with violent use in a longitudinal fashion).

Maximum thickness is often the same as bulbar thickness, as in most typical Levallois points. Exceptions to this general pattern include points whose butts are thinned to facilitate hafting, or are initially distally and medially thick. Similarly, maximum width is basal width only in “broad-base” points. For consistency, all metric attributes on the Aduma points in this study were directly collected by one of us (Y.S.) using digital calipers. Similarly, since artifact weight in Brooks *et al*. [2] was calculated indirectly, the present study directly measured all pointed pieces using a digital scale. Modification of the base of pointed artifacts was documented qualitatively, following procedures in Brooks *et al*.[2].

TCSA and TCSP were calculated in this study following the methods detailed by Sisk and Shea [7]. For TCSP, values from the more restrictive measure of triangular, rather than rhomboidal, cross section were used [7]. Dimensional measurements for morphometric analyses were collected on 100 pointed artifacts from the four localities. Twenty-two pieces were found to be unsuitable for this analysis because the measurements necessary for the calculations of TCSA and TCSP could not reliably be identified on them due to transverse fractures. TCSA and TCSP data on 100 Aduma points were statistically compared with data from ethnographic/archaeological dart tips and arrowheads, as well as experimental thrusting spears. Raw metric data for experimental spears were generously provided by John J. Shea. These experimentally produced thrusting spears were employed in a study conducted to assess the relationship between Levallois point morphology/size and the functional demand for stone spear points [15]. The comparative ethnographic/archaeological arrowheads and spearthrower dart tips are housed in the American Museum of Natural History and several other museums in the US. Since most arrowheads, but a smaller number of atlatl darts, were still attached in their hafts, armature type for these North American samples, most of which are less than a thousand years old, can be considered known. Measurements for these samples were collected by, and are available in Thomas [35] and Shott [36]. Raw metric data for the Aduma stone points employed in the present study are available as supporting information accompanying this paper. The independent sample t-Test has been employed to compare data on each assemblage with ethnographic spears, darts, and arrows, respectively, thus providing a powerful comparison of two sets of data. For larger sets of data with non-symmetric distribution and unequal variance, the Kruskal-Wallis H test is employed. This test represents the nonparametric alternative to the one-way ANOVA, testing whether the mean ranks of the groups are the same.

The macroscopic identification of artifact edge damage was conducted using a hand lens and intermittently a low-power (<20x) magnification on binocular reflected light microscope. Examination of microfractures on pointed artifacts focused on the identification of fracture wings, which represent a form of Wallner lines. Fracture wings are V-shaped, with their apex pointing toward the direction of fracture propagation. The angle of divergence of plane fracture wings on a given crack front allows the calculation of fracture velocity for an artifact of a raw material of a known distortional wave velocity. The higher the velocity of the impact fracture the narrower the angle of divergence of a fracture wing [37]. For the identification of microfracture features, this study examined points made on obsidian and siliceous rocks. A Keyence-600N microscope housed in the National Museum of Ethiopia, Addis Ababa, was used for this analysis.

## Results

### Point size and shape

**Fig 3:**
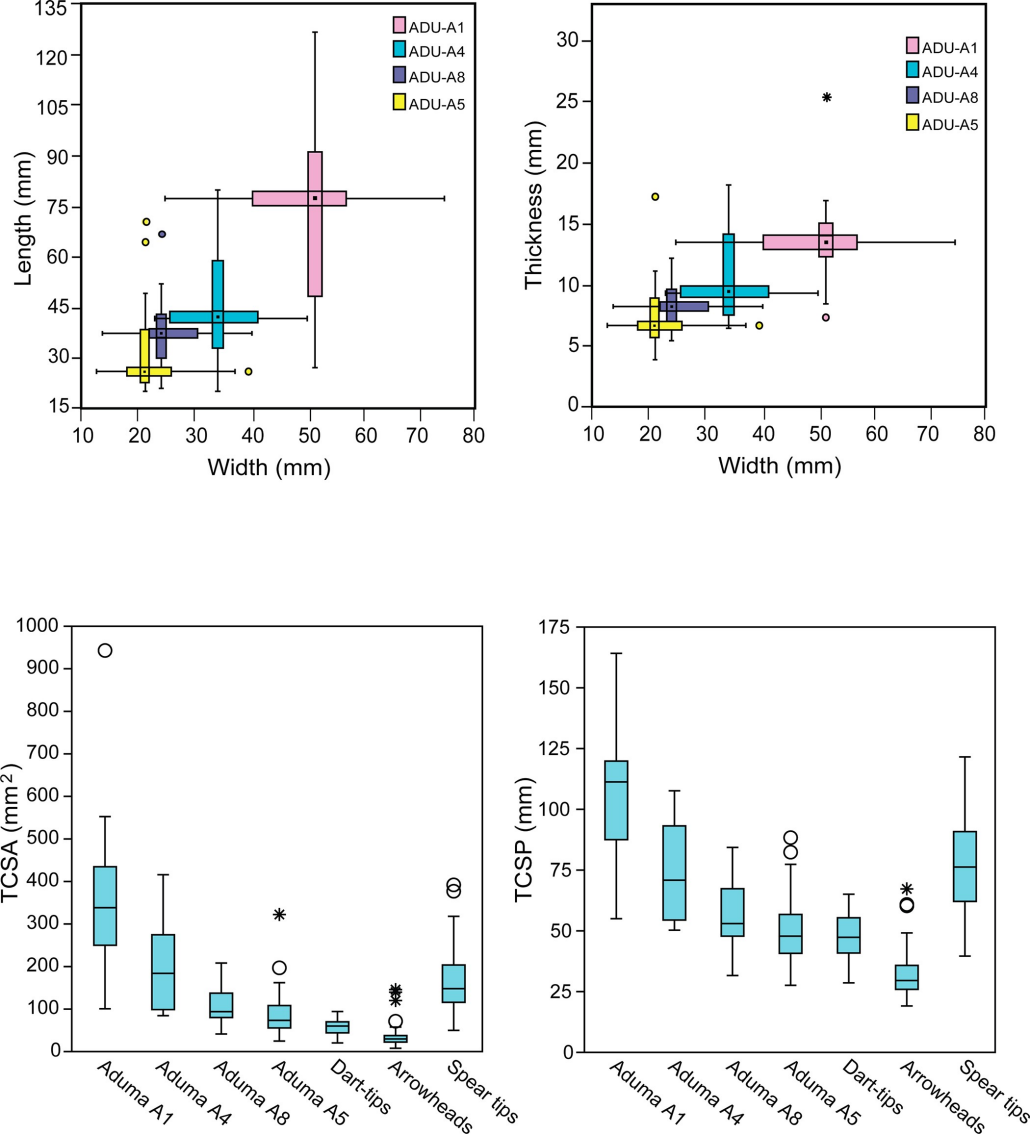
Box-plots of Aduma lithic point dimensions (top); Tip cross-sectional area (TCSA), and perimeter (TCSP) of Aduma and ethnographic point assemblages (bottom).

One of the most noticeable aspects of the Aduma lithic assemblages is the presence of large points (in addition to a small, triangular biface on obsidian) at Aduma A1, and the progressive diminution across the younger assemblages in the sequence. Directionality among the three upper Aduma localities was tested for statistical significance. Dimensional measurements of point assemblages from the excavated Aduma layers are summarized in Table 1 and Fig 3. Aduma A5, the youngest locality, contains the smallest pointed pieces on average while Aduma A4 and A8 retain medium-sized points (Fig 3). Point size does not necessarily indicate weapon type or mechanisms of delivery. Notwithstanding, the diminution of points (length, width, thickness) from Aduma 1 to the younger occurrences of Aduma A4, A8 and A5 is statistically significant. This pattern has previously led to the hypothesis that it occurred in response to the requirements for complex projectiles, rather than other factors [2]. Within the younger Aduma assemblages, Aduma A8 and A5 maintain much smaller points than A4 (Table 1), even though raw material and core reduction patterns do not differ dramatically. More interesting results emerge when one combines this pattern with morphometric data for the respective assemblages.

**Table 1:** Comparison of Aduma points with ethnographic arrowheads, dart tips, and spear tips using independent sample *t*-test. (Units in mm/mm^2^; SD = Standard Deviation).

Samples(*n*)	Length (Mean ± SD)	Width (Mean ± SD)	Thickness (Mean ± SD)	TCSA				TCSP			
				(Mean ± SD)	*vs*. arrowheads	*vs*. dart tips	*vs*. spear tips	(Mean ± SD)	*vs*. arrowheads	*vs*. dart tips	*vs*. spear tips
Arrowheads (*118*)	unavailable	15.09 ± 3.9	4.13 ± 1.3	32.53 ± 19.94				31.35 ± 8			
Dart tips (*40*)	unavailable	23.05 ± 4.4	4.96 ± 1	57.95 ± 17.8	0.0001			47.2 ± 8.86	0.0001		
Spear tips (*28*)	87.6 ± 18.92	36.39 ± 10.4	8.75 ± 2.48	167.98 ± 89.3	0.0001	0.0001		76.94 ± 21.22	0.0001	0.0001	
Aduma A1 (*18*)	74.84 ± 27.68	48.41 ± 14.51	13.51 ± 4.04	347.6 ± 196.4	0.0001	0.0001	0.0002	104.08 ± 30.21	0.0001	0.0001	0.0003
Aduma A4 (*22*)	46.92 ± 16.73	34.07 ± 8.89	10.7 ± 3.66	193.85 ± 108.2	0.0001	0.0001	0.6601	74.5 ± 19.56	0.0001	0.0001	0.63205
Aduma A8 (*35*)	36.75 ± 8.91	25.57 ± 6.15	8.36 ± 1.7	110.2 ± 44.7	0.0001	0.0001	0.0044	56.25 ± 12.55	0.0001	0.0017	0.0002
Aduma A5 (*25*)	32.31 ± 12.95	23.05 ± 6.85	7.54 ± 2.68	92.99 ± 61.9	0.0001	0.0024	0.0003	50.76 ± 14.87	0.0001	0.68088	0.0001

Based on TCSA values, the Aduma point assemblages can be classified into three groups. Aduma A1 points stand out as having statistically significantly larger average TCSA values than any of the recent ethnographic projectiles. In fact, the average TCSA of Aduma A1 points is twice as large as that of ethnographic spear tips (Table 1; Fig 3). Aduma A4 points have average TCSA values that do not differ significantly from ethnographic spear tips (*p* = 0.6601). Finally, Aduma A5 and A8 points have average TCSA values that are considered significantly bigger than those of ethnographic dart tips or arrowheads, but significantly smaller than ethnographic spear tips (Table 1). Comparisons of TCSP values provide a largely similar picture in which Aduma A1 is isolated by its significantly higher TCSP, while A4 retains average TCSP values statistically not distinguishable (*p* = 0.63205) from ethnographic spear tips. Most interestingly, the average of the Aduma A5 point assemblage falls within the range of ethnographic dart tips (*p* = 0.68088; Table 1; Fig 3).

Closely related to TCSA and TCSP, point angle (also referred to as penetrating or tip angle) represents yet another important attribute of ballistic technologies, as it affects not only the aerodynamics of projectiles, but also of penetration depth and impact [5]. Brooks *et al*. [2] have suggested that point angle was kept largely consistent among the Aduma point assemblages and that this could be in order to meet penetrative requirements in design. Table 2 provides summary statistics for Aduma point angle measurements by assemblage. Overall, the average point angle for all four of the Aduma assemblages is consistent at 53 to 64 degrees. However, minimum and maximum angle measures range relatively broadly between 32 and 94 degrees. Inter-assemblage difference in point angle measurements is also significant for all localities (Kuskal-Wallis test *H* = 10.51; *p* = 0.014). Aduma A5 maintains the smallest mean tip angle at 53°, while A4 has the largest. In what appears to be inconsistent with the other dimensional patterns, the Aduma A1 points maintain average angle very similar to A8.

**Table 2:** Aduma point attributes: tip angle, artifact weight, modification for hafting (through proximal thinning), and damage, including DIFs.

**Sample (*n*)**	Tip angle (Mean ± SD)	Weight (Mean ± SD)	% Proximal thinning	Damage count (Diagnostic Impact Fractures)
Aduma A1 (*18*)	58 ± 11.4	58.3 ± 42	38	2 (1: transverse fracture)
Aduma A4 (*22*)	64 ± 12.7	20 ± 18.8	68	6 (4: all transverse fractures)
Aduma A8 (*35*)	58 ± 12.5	7.36 ± 4.1	48	9 (2: 1 burin-like; 1 transverse)
Aduma A5 (*25*)	53 ± 10.7	6.8 ± 7.9	40	6 (4: 2 burin-like; 2 transverse)
**Sample (*n*)**	Tip angle (Mean ± SD)	Weight (Mean ± SD)	% Proximal thinning	Damage count (Diagnostic Impact Fractures)
Aduma A1 (*18*)	58 ± 11.4	58.3 ± 42	38	2 (1: transverse fracture)
Aduma A4 (*22*)	64 ± 12.7	20 ± 18.8	68	6 (4: all transverse fractures)
Aduma A8 (*35*)	58 ± 12.5	7.36 ± 4.1	48	9 (2: 1 burin-like; 1 transverse)
Aduma A5 (*25*)	53 ± 10.7	6.8 ± 7.9	40	6 (4: 2 burin-like; 2 transverse)

Much like overall size, the weight of stone points has long attracted the interest of archaeologists as a variable that can potentially separate between different armature types (e.g., [38,39]). Average estimated weights of 2.07 ± 0.28 and 4.38 ± 2.11 grams are reported for North American ethnographic and archaeological arrowheads (*n* = 132) and dart points (*n* = 10), respectively [35: 469]. However, since these values had to be calculated from other dimensions indirectly—for the points were still hafted—and since the sample size for dart points is extremely small, the potential of weight as a reliable discriminator between armature types remains unclear. Considerable overlap in the weight of stone points used to tip different weapon types further limits its applicability as a good discriminator [40]. Notwithstanding these limitations, we measured the weight of the Aduma stone point assemblages.

Table 2 shows that within the Aduma point assemblages, the weight of pointed artifacts decreases progressively across the succession. It is particularly interesting that the Aduma A5 assemblage maintains the smallest artifact weight at an average of 6.8 g—a value strikingly similar to the estimate from indirect calculation provided by Brooks and colleagues [2], and within the range of Thomas’s [35] sample of dart points. At an average weight of 7.4 g, Aduma A8 has only a slightly higher average weight than A5. On the other hand, Aduma 4 points are on average almost three times as heavy (at 20 g) as A5 and A8; Aduma A1 maintains the highest average values of point weight at 58 g. Weight, therefore, separates the Aduma points into three distinct categories: A1 with average weight perhaps outside the range of even hand-cast spear tips; A4 with average weight within the range of hand-cast spears; and A8 and A5 with average weight similar to dart tips. Examination of the individual data (Supp. Info. S1 Table) suggests that the A4 weight distribution is bimodal, with about a third of the assemblage falling within the ethnographic dart tip range, while more than half fall in the same range as the A1 sample. Such a pattern suggests that more than one weapon system may be represented in the sample. The A8 and A5 sample distributions, however, are unimodal and dominated by points with weights in the ethnographic dart tip range, with a much smaller tail towards the larger values.

### Impact fracture

**Fig 4:**
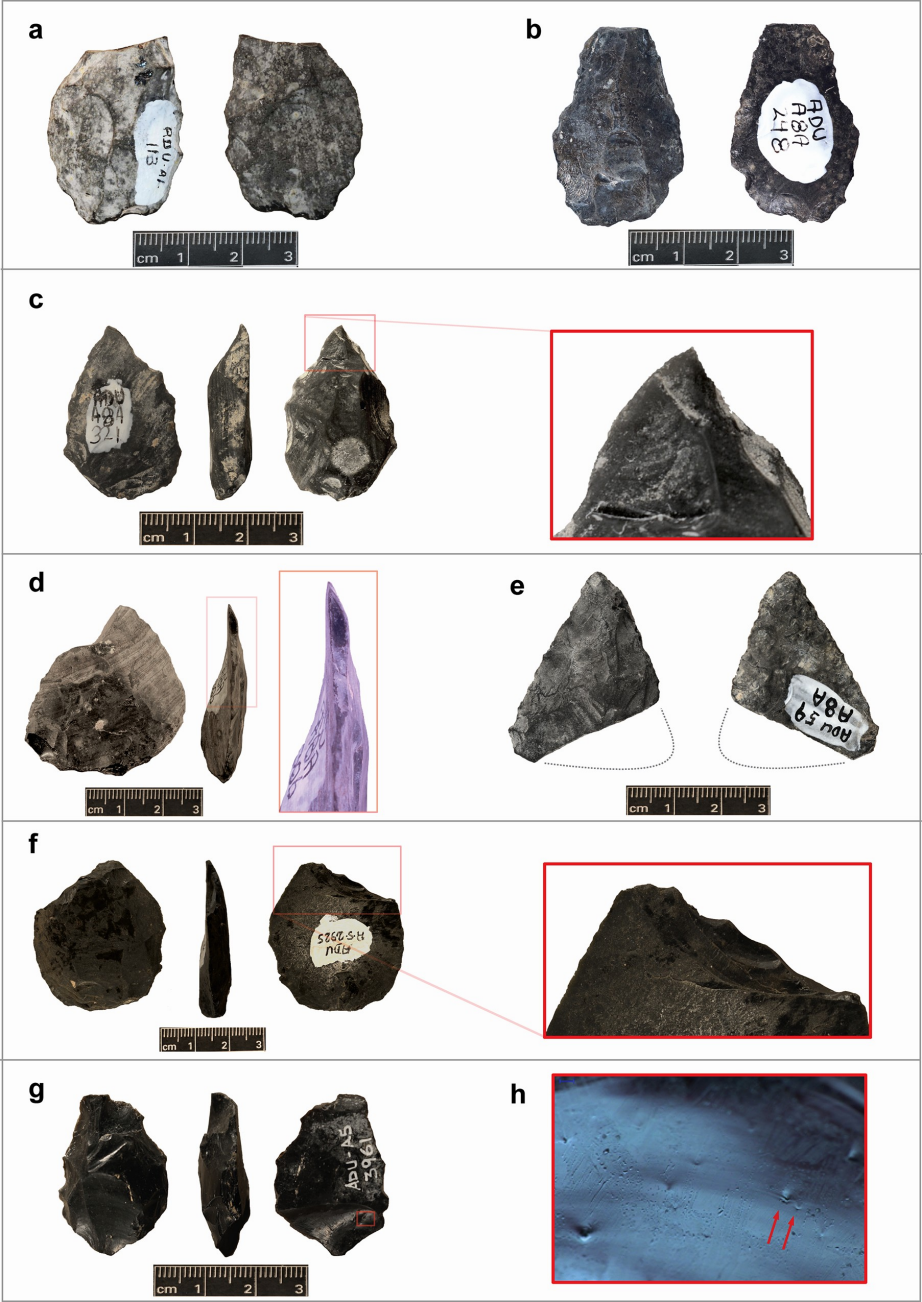
Selected examples of impact fractures on Aduma points: (*a*) transverse fracture with snap termination on the smallest of Aduma A1 points; (*b*) tip snap on point from A8; (*c*,*d*) burin-like fractures on points from A8; (*e*) point with transversely fractured base from A8; (*f*) bending fracture with hinge termination across the distal tip of an A5 point, with fracture occurring after intentional retouch; (*g*) burin-like fracture on the distal tip of an A5 point—note also the damage across the proximal end on the ventral face of this point; (*h*) photomicrograph of fracture wings along the proximal end of a piece from Aduma A5 depicted in “*g*”.

The count of damaged points in the Aduma assemblages suggests yet another distinct pattern. In total, 23% of the analyzed Aduma points exhibit impact-related damage. The Aduma A1 assemblage contains only two points with macrofractures, one of which is a tip fragment not included in the morphometric aspect of this present study. About a quarter of points in the Aduma A8 and A4 assemblages, and over a third of the A5 points exhibit macrofracture damage. Out of the damaged points, about half exhibit impact fractures commonly considered diagnostic of use of stone points as hunting armature tips (i.e., DIFs). One of the two damaged Aduma A1 points particularly shows a transverse fracture on the distal tip. Aduma A8 has nine impact fractured points, of which two can be categorized confidently as DIFs. The A4 and A5 point assemblages contain four out of six pointed pieces with DIFs each. Table 2 summarizes the count of DIFs per locality, while Fig 4 provides examples of pointed pieces with tip damage, including DIFs.

Even though more than half of the Aduma points analyzed here are made on obsidian, most of these show a high degree of patination, making the microscopic examination of impact-induced fractures largely difficult. Out of 68 obsidian points (including distal fragments not incorporated into the metric analysis), only 19 have fresh fracture fronts suitable for microfracture analysis. Of these, only a single impact damaged point from Aduma A5 exhibits a clear microfracture feature in the form of a fracture wing in a relatively large fracture front across the base of an obsidian point (Fig 4). At ~130 degrees angle of divergence, this fracture wing on an obsidian point from Aduma A5 is fairly acute, and could therefore indicate relatively violent impact [32,33].

Calculation of the actual instantaneous fracture velocity for this piece has been, however, difficult due to several reasons. First, obsidian provenance data required for matching the specific artifact of interest to a particular obsidian source is exceptionally complicated for Aduma. Out of nine different obsidian groups identified in the previously geochemically characterized Aduma A8 assemblages, only one source has been positively matched; the remaining eight obsidian sources exploited by the Aduma early humans are as yet unknown [41]. It is important to note here that even for Aduma A8, only two out of the 20 analyzed artifacts were possibly linked to the Ayelu source, based on comparisons of the most diagnostic elements (Zn, Rb, Sr, Y, Zr Nb and Th) from an earlier geological study [42]. The earlier Ayelu obsidian analysis also measured TiO_2_, Fe_2_O_3_, and MnO, but comparison with the two Aduma samples on this ground is not conclusive, making the Ayelu identification very tentative. Such a pattern contrasts with other localities of Late Pleistocene age nearby, such as Upper Halibee, where eight out of 15 geological sources have been matched with obsidian artifacts [41].

Our latest attempt to characterize this A5 piece using portable XRF has yielded dramatically different chemical composition, thus making the calculation of the physical properties impossible. Similarly, the presence currently of only limited experimental data on the instantaneous fracture velocities and corresponding loading rate regimes—and weapon delivery mechanisms—further complicate potential comparisons and inferences. Finally, since the microfracture feature on the singular Aduma A5 piece is associated with a proximal, rather than distal, tip impact fracture (most likely as a result of violent crushing of the base while the piece was still in the haft), the exact implication for weapon delivery mode of a velocity measure from such an ‘indirectly’ initiated instantaneous fracture would have further been difficult to establish.

## Discussion

Current inferences based on circumstantial lines of evidence suggest that complex projectiles may have originated in Africa sometime between 50 and 100 ka [6,7]. However, the archaeological evidence in hand has not adequately answered questions relating to the timing and trajectory of the development of complex projectiles. The consensus view seems to favor the adoption, first in Africa, of spearthrowers and their later replacement by the bow and arrow technology [43]. Substantial archaeological evidence for composite projectiles—microlith-barbed osseous points—comes only from the Upper Paleolithic and is interpreted as propelled by spearthrowers [44,45]. Archaeological and ethnographic evidence shows that these weapon systems were used by hunter-gatherers side by side. Considering the advantages and limitations of each of the ethnographically known complex projectile systems (i.e., the bow-and-arrow, and spearthrower-and-dart), the trajectories of their adoption and implications of these remain inadequately addressed.

The suggestion for the evolution of complex projectiles in the early Late Pleistocene at Aduma is particularly interesting due to the antiquity and succession of these archaeological occurrences, and the multiplicity of attributes collected on >100 stone points from four distinct contexts, some in spatial association with an anatomically modern human cranium [10,34].

Converging lines of morphometric and macrofracture evidence now suggest that complex projectile use at Aduma may have occurred at the younger end of the documented early Late Pleistocene succession. The oldest Aduma points from locality A1 retain significantly greater average dimensions as well as larger TCSA and TCSP values than even ethnographic spear tips. On the other hand, average dimensions of points from Aduma A4 fall within the variation of ethnographic spear tips, both in TCSA and TCSP, but the bimodal nature of the distribution could reflect the presence of two different weapon systems, possibly both spears and spearthrowers. Aduma A8 points on average are significantly smaller than the ethnographic spear tips, but larger than the ethnographic dart tips. Finally, Aduma A5 points are statistically indistinguishable on average from ethnographic dart tips in TCSP. This pattern suggests that, as far as overall point dimension and tip morphometric attributes, Aduma A4 may be older than A8. This picture is particularly intriguing as the true stratigraphic relationship between these two localities is as yet undetermined. Furthermore, if the ethnographic sample accurately mirrors past weapons systems, a) arrow armatures may not have been adopted during the entire sampled succession represented at Aduma, and b) spearthrower darts emerged during the younger phase of the Aduma early Late Pleistocene succession, possibly as a small component at Aduma A4, probably at Aduma A8, but more conclusively at Aduma A5. The latter inference is specifically interesting as TCSP has been experimentally shown to be a particularly consistent indicator of a point’s penetrative quality [26], and because this study employed the more restrictive triangular measure of TCSP [7].

A point’s distal tip angle affects penetrative ability of projectile armatures and the resistance of the point to breakage during impact. The lack of data on the tip angles of the North American ethnographic and archaeological arrowheads and dart tips limits assessment of the applicability of this variable in the identification of prehistoric projectile systems. Average penetrating tip angles for Upper Paleolithic points (from France) considered as tips of complex projectiles reach up to 49 ± 12 degrees [46]. With an average point tip angle of 53 ± 10 degrees, Aduma A5 has the closest measures to these data from archaeological points interpreted as composite tools. Once again, the interesting aspect of point tip angle in the Aduma assemblages is that there is a general consistency, with noticeable reduction in A5. As in Brooks *et al*. [2], point angle seems to have been generally maintained and is less variable within each Aduma assemblage. Also, across assemblages, point angle is consistent, averaging ~58 degrees. However, unlike the other morphometric measures applied here, tip angle does not provide a clear pattern of change at Aduma, with the oldest assemblage of Aduma A1 showing relatively small and more consistent point angle than younger assemblages in the succession. Considering the lack of any strong support from other parameters for the use of Aduma A1 points for projectile weapons, functional requirements other than use as spear tip (e.g., use as knife) may have necessitated specific responses in the form of overall narrow point angle in this assemblage. At any rate, the youngest assemblage of Aduma A5 maintains a comparably small and consistent point angle, perhaps this time in response to the constraining requirements of use as dart tips.

The weight of projectiles represents one of the crucial traits of such technologies, as this determines the amount of draw force required to effectively propel them, and their effective impact range. Consistent with ethnographic cases where hunters carry only a limited number of hafts, Brooks *et al*. [2] highlight maintainability as an important aspect of prehistoric projectile technologies as well. Hafting requirements were, therefore, not only a potential constraint on point dimension and proximal shape, but also on point weight. If only a limited number of similar hafts were used by prehistoric hunters, as in ethnographic examples of hafted technologies [47], then points meant to be hafted in replacement should also maintain weights considered ideal for the specific projectile system. An intriguing diachronic trend in the Aduma early Late Pleistocene succession toward complex projectiles comes from the weight of pointed pieces. From the oldest Aduma A1 through to the youngest A5 assemblages, artifact weight shows progressive reduction. This study could not find adequate and reliable data on the effective weight of stone points considered suitable to function as tips of specific types of complex projectiles. Thomas [35: 469] reports the mean weight of archaeological dart points (*n* = 10) to be 4.38 grams (ranging between 1.6 and 7.9 grams). With an average weight as small as 6.8 g, the Aduma A5 (and possibly A8) points seem to be conceivably already small enough to tip darts. However, because the sample size for ethnographic dart points is rather too small, and experimental data on the effective weight for suitable projectile points unavailable, the strength of the pattern observed in the weight Aduma points remains difficult to assess any rigorously. Better conclusions can be made about the substantially smaller estimated weight (average = 2.07 g) of arrow tips, for which there is a very large ethnographic and archaeological sample [35: 469].

The Aduma early Late Pleistocene occurrences are distributed across an aggrading context of sediments deposited atop erosional surfaces. Such inferred alterations in landform and resource availability, including lithic raw material, have been interpreted as necessitating new forms of adaptive strategies by denizens in order to cope with the changes. It is particularly interesting to note the presence, for the first time in the succession, of minimally water-dependent bovids like oryx at Aduma A5, as well as higher numbers of savanna to woodland adapted species of bovidae [34]. Study of the mammalian faunal samples from the Aduma localities also noted, however, that the faunal remains were affected in major ways by both aggradational processes and *in situ* post-depositional destructive taphonomic processes such as calcrete infiltration into bones and teeth and removal of cortex. These processes hampered adequate testing of both environmental hypotheses and identification of potential hunting impacts [19]. Future work in these Aduma contexts and their contents promises a clearer understanding of the development of complex projectiles regionally as well as globally, including relevant ecological and social/demographic contexts potentially affecting such decisive technological transitions.

## Conclusions

Our assessment of the Aduma point assemblages supports earlier suggestions that spearthrower darts emerged at least by the end of the early Late Pleistocene [2], and adds to a growing interest in the early sophistication of stone-tipped projectiles. Irrefutable evidence for early complex projectiles is as yet lacking from much of Africa prior to 40 ka [5]. Claims of complex projectiles from *later* Late Pleistocene contexts benefit from the preservation of multiple lines of circumstantial evidence, including residues and associated fauna. For contexts with large faunal samples, such as Porc-Epic Cave, the integration of data from weapon-impact marks on bone [19] with those from lithic assemblages [7] and related faunal exploitation data [23] promises new avenues of research. Because projectile data are extremely rare from sub-Saharan early Late Pleistocene occurrences, and because this period is critical to our understanding of modern human origins and dispersal, a cautious interpretation of the limited lines of evidence in hand is necessary [48]. Archaeologists must strive to employ in their studies multi-stranded approaches to the identification of projectile weaponry. Care must also be taken not to confuse evidence for hafting and hunting with hand-delivered weapons with that using ranged ones. Where possible, it is necessary to distinguish between the different categories of ranged weapons, from hand-cast spears (*simple* projectiles) to *complex* one—darts propelled by spearthrowers and arrows shot with the aid of a bow. In eastern Africa, more experimental studies with widely exploited obsidian sources (such as that of K’one) promise to widen the applicability of the relatively robust and objective impact fracture velocity method [41]. At Aduma, additional obsidian provenancing and experimental work should test the general pattern suggesting the use of some of the pointed pieces in the A5 assemblage as complex projectiles.

## Supporting information

S1 Table Metric data for the Aduma points discussed in the present study. (XLSX)
